# Post-Transcriptional Gene Silencing of Glucanase Inhibitor Protein in *Phytophthora cinnamomi*

**DOI:** 10.3390/plants12223821

**Published:** 2023-11-10

**Authors:** Patrick Ferreira, Abdessalem Chahed, Letícia M. Estevinho, Natália Seixas, Rodrigo Costa, Altino Choupina

**Affiliations:** 1 Centro de Investigação de Montanha (CIMO), Instituto Politécnico de Bragança, Campus de Santa Apolónia, 5300-253 Bragança, Portugal; patrickppferreira@gmail.com (P.F.); al@ipb.pt (A.C.); natalia.seixas@ipb.pt (N.S.); ao@ipb.pt (R.C.); albracho@ipb.pt (A.C.); 2 Laboratório para a Sustentabilidade e Tecnologia em Regiões de Montanha, Instituto Politécnico de Bragança, Campus de Santa Apolónia, 5300-253 Bragança, Portugal

**Keywords:** chestnut, *Phytophthora cinnamomi*, ink disease, *GIP*, RNA interference, subcellular localization

## Abstract

Ink disease is considered one of the most significant causes contributing to the decline of chestnut orchards. The reduced yield of *Castanea sativa* Mill can be attributed to two main species: *Phytophthora cinnamomi* and *Phytophthora cambivora*, with the first being the main pathogen responsible for ink disease in Portugal. *P. cinnamomi* is a highly aggressive and widely distributed plant pathogen, capable of infecting nearly 1000 host species. This oomycete causes substantial economic losses and is accountable for the decline of numerous plant species in Europe and worldwide. To date, no effective treatments are available to combat these pathogens. Given chestnut’s economic and ecological significance, particularly in Portugal, it is crucial to investigate the molecular mechanisms underlying the interaction between *Phytophthora* species and host plants. This can be achieved through the study of the glucanase inhibitor protein (GIP) produced by *P. cinnamomi* during infection. The technique of RNA interference (RNAi) was employed to suppress the *GIP* gene of *P. cinnamomi*. The resulting transformants, carrying the silenced gene, were used to infect *C. sativa*, allowing for the assessment of the effects of gene silencing on the plant’s phenotype. Additionally, bioinformatics tools predicted the secretion of GIP protein. The obtained results validate RNAi as a potential alternative tool for studying molecular factors and for controlling and managing *P. cinnamomi*.

## 1. Introduction

Ink disease is a highly destructive ailment that affects *Castanea sativa*, causing root and collar rot in adult trees as well as seedlings in nurseries, plantations, and forests [1]. Symptoms of the disease in mature trees include chlorotic leaves, crown thinning, and the presence of immature bark adhering to the tree after leaf fall. The root system exhibits extensive necrosis of the taproot, which extends to the lateral roots and reaches several centimeters up the stem. Infected plants also display a rapid or gradual withering of the leaves [2]. The distribution of ink disease corresponds to the presence of *Phytophthora cinnamomi* and *Phytophthora cambivora*, the two main pathogens responsible for the disease. Typically, these species lead to the death of adult trees within one to three years [2].

*P. cinnamomi* can persist as a saprophyte in the soil for extended periods, capitalizing on favorable conditions to produce abundant asexual, biflagellate zoospores. These motile zoospores are attracted to susceptible infection sites, where they attach and invade the plant. Within a few days, the hyphae spread throughout the tissues of vulnerable plants, forming sporangia on the plant surface and rapidly increasing the disease inoculum. *P. cinnamomi* has been found to survive for up to six years in moist soil, with moisture playing a crucial role in the establishment, spread, and longevity of *P. cinnamomi* diseases. A liquid environment is essential for asexual sporulation, including the formation of sporangia and the release and activity of motile zoospores. In line with this, disease development is particularly enhanced after heavy rainfall and in waterlogged soils [3,4,5].

Traditional fungicides are ineffective against *Phytophthora* diseases due to the significant evolutionary distance between *Phytophthora* and true fungi. Likewise, chemical compounds that inhibit fungi may have no effect on *Phytophthora*. Therefore, alternative approaches are necessary to control *Phytophthora* diseases, as these root pathogens can persist as chlamydospores in the soil and in the roots of asymptomatic plants [3].

Recent research has provided valuable insights into the molecular mechanisms underlying pathogenicity and the eradication of diseases in plants [6], such as those caused by this pathogen [1,7,8,9,10,11]. The interaction between plants and pathogens involves the secretion of glucanase inhibitor proteins (GIPs) by the pathogen in response to the hydrolytic proteins of the host plants, specifically endo-β-1,3-glucanases [12,13]. Given the significant role of GIPs in pathogenicity and the response of the host plant, it is crucial to understand the mechanism of action of these proteins.

The *GIP* gene of *P. cinnamomi* (GenBank, NCBI, accession number AM259384) has a total size of 1171 bp and an open reading frame (ORF) of 810 bp and encodes 269 amino acids. The gene amplification was accomplished using generic primers designed based on the homology of *GIP* ORF in *Phytophthora sojae* and the TAIL-PCR technique [14]. The alignment of the *P. cinnamomi GIP* sequence with sequences from *P. sojae* and *P. infestans* revealed significant homologies. In these pathogens, *GIP* binds to and inhibits the activity of plant extracellular endoglucanases involved in defense responses, thereby suppressing the degradation of glucan in oomycete cell walls and preventing the release of glucan elicitors and the subsequent defense responses. However, the biological significance of GIP proteins as suppressors of endoglucanases is not yet fully understood. Several molecular-level questions remain, including the identification of domains and key residues of the inhibitor proteins that contribute to the specificity of recognition [15,16].

The primary objective of this study is to enhance our understanding of the molecular interactions between *P. cinnamomi* and *C. sativa* by investigating the impact of pathogen gene silencing on the host phenotype and studying the subcellular localization of the GIP protein involved in the infection mechanism. This knowledge is crucial for the development of effective control strategies.

## 2. Results

### 2.1. Cloning the GIP-Silencing Cassette into the pUC57 and pTH210 Vectors

**Figure 1:**
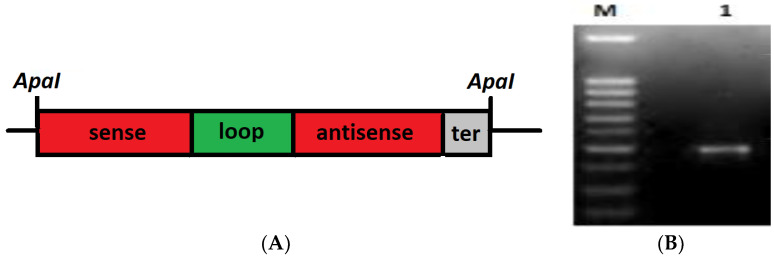
(**A**) Structure of the *GIP*-silencing cassette. (**B**) Visualization of the synthesized cassette on agarose gel (M) DNA ladder of 100 bp; (1) the *GIP*-silencing cassette (551 pb).

The *GIP* gene-silencing cassette (Figure 1) was constructed in a gradual way by successive ligations, with T4 DNA ligase, of the constituent fragments, initially in the *E. coli* pUC57 vector.

**Figure 2:**
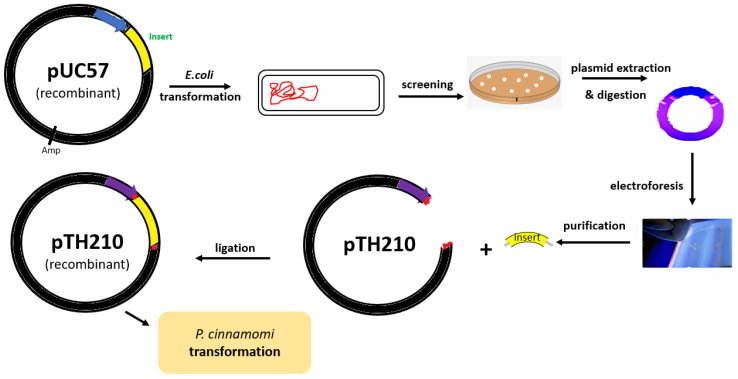
Construction and cloning strategy of the *GIP* gene-silencing cassette.

The binding confirmations of the respective fragments were confirmed by enzymatic digestions visualized in electrophoresis and by plasmid DNA sequencing with the cloned fragments in each step and according to the schemes in Figure 2.

### 2.2. Confirmation of P. cinnamomi Transformation by Culture in a Medium Containing Hygromycin Antibiotic

**Figure 3:**
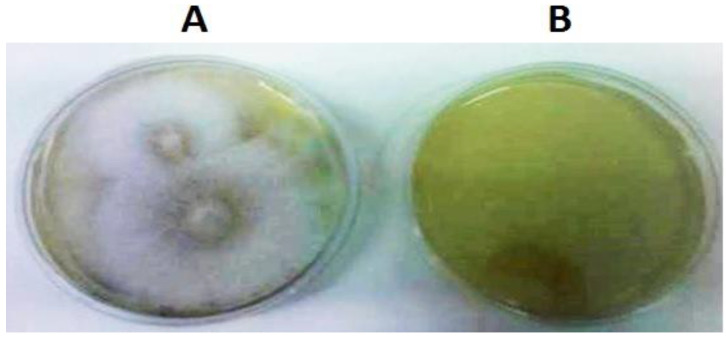
Growth of transformed *P. cinnamomi* (**A**) and native *P. cinnamomi* (**B**) in medium containing hygromycin.

The recombinant plasmid pTH210, with the *GIP*-silencing cassette, was used to transform *Phytophthora cinnamomi* by electroporation; then, the transformants were cultivated on a medium containing hygromycin (200 μg/mL) and incubated for ten days at 25 °C in the dark. At the same time, non-transformed *P. cinnamomi* were incubated in the same conditions as negative control plates in medium containing hygromycin (200 μg/mL). After ten days of incubation at 25 °C in the dark, there was no growth of the negative control of *P. cinnamomi* in the medium containing the hygromycin selection marker, while for the transformed *P. cinnamomi*, only six out of ten grew in the medium with hygromycin (Figure 3).

The growth of transformed *P. cinnamomi* in medium containing hygromycin means that they can produce the hygromycin phosphotransferase (HPT) protein that degrades the hygromycin antibiotic, giving resistance to these microorganisms. That is only possible with the transformation of *P. cinnamomi* by the pTH210 vector, meaning that this transformation was successful.

### 2.3. Genotypic Confirmation of the P. cinnamomi Transformation

**Figure 4:**
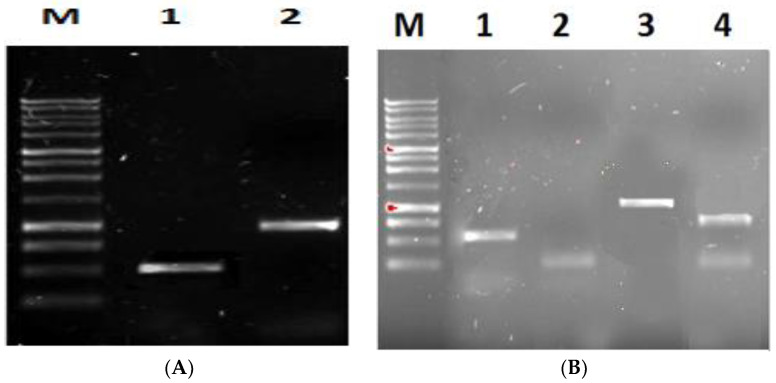
(**A**) PCR amplification of 551 bp corresponding to the *GIP*-silencing cassette sequence (1) and the region of 1090 bp within the hygromycin gene (*HPT*) (2). (**B**) Digestion of the *GIP*-silencing cassette of 551 bp with the enzyme MseI (1 and 2); digestion of the 1090 bp hygromycin (3) with the enzyme PstI (4).

To confirm the transformation of *P. cinnamomi* by PCR screening, the genomic DNA of transformed *P. cinnamomi* was extracted after ten days of incubation, and specific primers were designed to amplify a region of approximately 551 bp corresponding to the *GIP*-silencing cassette sequence and another region of 1090 bp within the hygromycin gene (*HPT*). With these procedures, it is expected to prove the integration of the recombinant plasmid pTH210 in the genome of *P. cinnamomi* (Figure 4A). The amplification by PCR of the *GIP*-silencing cassette produced a band of approximately 551 bp, while the amplification of the hygromycin segment produced a band of 1090 bp. The two PCR products were purified and digested by restriction enzymes to confirm the fragment’s identity. After visualization on an agarose gel, the digestion of the *GIP*-silencing cassette fragment of 551 bp with the enzyme MseI generated two bands: one of 284 bp and another of 267 bp. The digestion of the 1090 bp hygromycin fragment was performed with the enzyme PstI and generated a band of 282 bp and another of 808 bp (Figure 4B).

After digestion of the cassette fragment with the MseI enzyme, the two generated bands were superimposed (284 bp and 267 bp). These results confirm that the pTH210 recombinant plasmid was integrated into the *P. cinnamomi* genome.

### 2.4. Sequencing of the Transformed Phytophthora

To confirm the integration of the recombinant pTH210 vector in the genome of *P. cinnamomi*, the PCR products of the hygromycin fragment and the *GIP*-silencing cassette were sequenced and then analyzed with the BioEdit program.

After that, the nucleotide sequences were blasted using the NCBI database to investigate the IDs of the sequenced genes. The alignment of similar genes was analyzed and compared using the Muscle Tool Server (CLUSTAL multiple sequence alignment) from the EMBL-EBI database.

The alignment results of the hygromycin fragment sequence showed a high similarity with the corresponding gene. However, for the *GIP* cassette sequence, only the initial portion of the sequence corresponding to the sense strand of the silencing construct exhibited complete homology with the *P. cinnamomi GIP* gene. This is because the construction of the cassette was solely based on the selected sense sequence within the ORF of the *GIP* gene sequence. The antisense sequence is inverted in comparison to the gene sequence, and the loop does not display any homology with the *GIP* gene sequence. These findings confirm the integration of the silencing cassette into the genome of *P. cinnamomi*. More information about the sequencing process will be available in the Supplementary Materials.

### 2.5. C. sativa Infection with Transformed and Controlled P. cinnamomi

**Figure 5:**
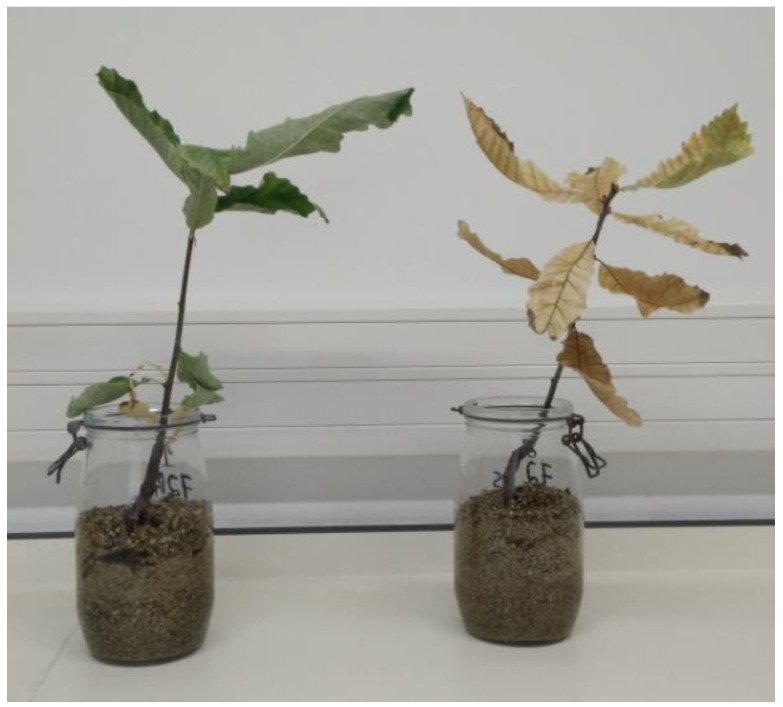
The infection of chestnut roots with transformed (**left**) and non-transformed *P. cinnamomi* (**right**).

Plants of *C. sativa* were sterilized and germinated in sterile vermiculite and then grown in a greenhouse until their root length reached approximately 30 cm. Three of these plants were infected with transformed *P. cinnamomi* mycelium with the *GIP*-silencing cassette, while the other three were infected with non-transformed mycelium. The roots of chestnuts infected by transformed and non-transformed *P. cinnamomi* were grown at room temperature in bottles with sterile vermiculite for 72 h. After the end of incubation, a morphological analysis of the chestnuts was performed to assess the effect of *GIP* gene silencing on the plants (Figure 5).

The infection of chestnut roots with transformed and non-transformed *P. cinnamomi* provided a comparative morphological analysis of the chestnut plants. The results demonstrate a clear contrast between chestnuts infected with transformed *P. cinnamomi* and those infected with non-transformed *P. cinnamomi.*

**Figure 6:**
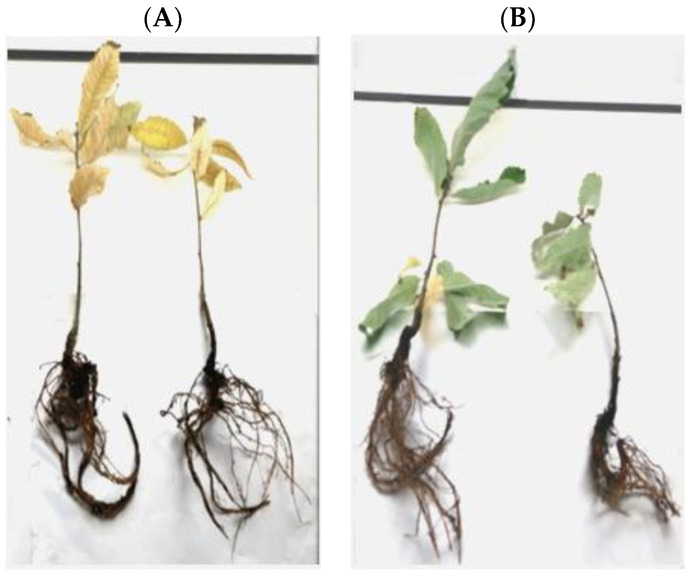
Seventy-two hours of infection of *Castanea sativa* with (**A**) native *P. cinnamomi* and (**B**) transformed *P. cinnamomi*.

After 72 h, two of the three chestnuts that have been infected by the transformed *P. cinnamomi* (transformed with the *GIP*-silencing cassette) revealed a smaller percentage of wilting leaves and root necrosis, while for plants infected with the non-transformed *P. cinnamomi*, there was a higher percentage of wilting leaves and root necrosis. As the *GIP* gene is considered responsible for the suppression of plant defense responses, enhancing susceptibility to disease symptom development [17], these results suggest the effectiveness of *GIP* gene silencing by the cassette cloned into *P. cinnamomi* (Figure 6).

### 2.6. Prediction of the Subcellular Localization of the GIP Protein

Several publicly available programs (CELLO, LOCtree, EuK-mPLoc2.0, Esl pred and SignalP 3.0) were used for the protein localization prediction. Regarding GIP, all tools (with different prediction methods) indicated an extracellular destination (a signal peptide located between positions 31 and 32 of the sequence).

## 3. Materials and Methods

### 3.1. Biological Materials

#### 3.1.1. *Phytophthora cinnamomi*

*Phytophthora cinnamomi* was isolated from soil samples associated with *Castanea sativa Mill* trees affected by ink disease in the Trás-os-Montes region (northeast of Portugal), characterized by molecular methods, and deposited in the Spanish Type Culture Collection (CECT) with the CECT 20919 code (isolated by Branco-Choupina, A. Culture media: 54/68; growth temperature (in °C): 25; incubation time: 7 days; atmospheric needs: aerobic) For more details, see https://www.es/uv.es/uvweb/uvweb/coleccion-espanola-cultivos-tipo/es/cect/catalogo-cepas/medios-cultivo/buscador-cepas-1285892802374.html (accessed on 6 April 2020).

#### 3.1.2. *Escherichia coli*

The NZY5α competent *Escherichia coli* cells (NZYTech, Lisboa, Portugal), which have similar properties to DH5α, were used to prepare the genetic constructions. The NZY5α competent cells allow for high-efficiency transformation in a wide variety of applications.

#### 3.1.3. *Castanea sativa* (Chestnuts)

Chestnuts that were one year old were used. The chestnuts were stratified beforehand in sand and kept in humid conditions at a temperature of 24 °C to promote germination. The germinated chestnuts were then planted in alveoli trays measuring 6 cm × 6 cm × 20 cm to facilitate normal growth.

#### 3.1.4. Culture Media

Solid media were prepared by adding agar to the corresponding liquid media. All media were prepared in ultra-filtered water and autoclaved at 120 °C for 20 min. To grow *E. coli* cells after transformation, Luria Bertani (LB) broth was prepared as follows: 1% (*w*/*v*) of tryptone, 1% (*w*/*v*) sodium chloride, and 0.5% (*w*/*v*) of yeast extract. To select clones resistant to antibiotics, media were supplemented with the corresponding antibiotic in a suitable concentration (100 μg/mL) after cooling. Then, the solution was aliquoted into sterile bottles, 5 mL in each; covered with the lid; and stored at room temperature. To culture *P. cinnamomi*, the following media were used: V8 liquid, V8 solid, YEPD (Yeast Extract Peptone Dextrose Medium), and PDA medium. V8 medium was prepared by adding 330 mL of V8 juice (Campbell Soup Co, Camden, NJ, USA) and 4.5 g of calcium carbonate (CaCO_3_) and by stirring for 30 min. Culture media were then centrifuged at 2500× *g* for 15 min at 20 °C. The cleared V8 juice (V8 broth) was then diluted 10-fold with ultra-filtered water and autoclaved. To prepare solid V8, 15 g of agar per 1000 mL was added to the diluted V8 broth before autoclaving. The liquid YEPD medium was prepared by mixing yeast extract 1%, peptone 2%, and D-glucose 2%; 2.0% agar was added to the solid YEPD. The potato dextrose agar (PDA, HIMEDIA, Einhausen, Germany ) was prepared by adding 39 g of commercial PDA powder to 1 L of ultra-filtered water.

#### 3.1.5. Culture Conditions and Maintenance of Microorganisms

Routine incubation temperatures were 25 °C for *P. cinnamomi* and 37 °C for *E. coli*. The cultures in liquid medium were generally grown under 150–200 rpm agitation in an orbital shaker (Stuart^®^, S150, Vernon Hills, IL, USA). Additionally, to maintain viable strains for a long time, the microorganisms were stored in glycerol (15–30%) at low temperatures of −70 °C. The bacterial strain used throughout this work was stored at −70 °C in 2 mL cryovials with 1/3 volume glycerol 100%. After being subcultured in Petri dishes. *E. coli* was stored at a temperature of 4 °C.

Two vectors were used in this study: pUC57 and pTH210. The pUC57 has 2710 bp and contains the ampicillin resistance gene. This plasmid was used for cloning the *GIP*-silencing cassette and therefore transformed *E. coli* to propagate the construct. On the other hand, pTH210 has 5030 bp, is driven by the *Hsp70* promoter, and contains the hygromycin resistance gene. This vector was used to insert the *GIP*-silencing cassette into the genome of *P. cinnamomi.*

### 3.2. DNA Extraction, Isolation, and Purification

To procure the genomic DNA of *P. cinnamomi*, the oomycete was cultivated on a PDA medium, shielded with aluminum foil, and maintained at 25 °C for six days. The DNA extraction process employed the following components: a lysis solution comprising 200 mM Tris-HCL, 25 mM EDTA, 250 mM NaCl, and SDS at 0.5% (*w*/*v*). Subsequently, deproteinization was carried out using a mixture of phenol, chloroform/isoamyl alcohol (25:24:1), and DNA precipitation through ethanol washing at −20 °C. The resultant DNA pellet was reconstituted in ultra-filtered water. To ensure the purity of the extracted DNA, RNase at a concentration of 5 mg/mL was added and incubated at 37 °C for 5 min [1].

To assess and refine the DNA fragments, electrophoresis was conducted in a 0.8% (*w*/*v*) agarose gel within TAE buffer (Tris-Acetate 40 mM, 1 mM EDTA), supplemented with 0.5 μg/mL of GreenSafe Premium (NZYtech, Lisbon, Portugal). The electrophoresis was performed for 40 min at room temperature under 80 V [1]. Subsequently, by using the ChemiDoc™ XRS+ imaging system, 6.1 Software for Windows (BioRad, Hercules, CA, USA), the gel was irradiated to visualize and determine the size and intensity of the target DNA band by comparing it to DNA ladders.

The isolation and purification of DNA fragments were achieved by excising the agarose band containing the desired fragment from the gel prepared in TAE using a sterile razor blade. DNA molecules within each band were further purified using the QIAquick^®^ Gel Extraction Kit (QIAGEN, Hilden, Germany), following the instructions provided by the manufacturer.

### 3.3. Design of a Silencing Cassette (shRNA-Based Vector) for GIP Gene

Several techniques can be employed to construct silencing cassettes, such as annealed oligonucleotides and PCR-based cloning. The PCR-based approach was chosen due to its cost-effectiveness and enhanced reliability. The silencing shRNA construct was created by connecting a 5′-sequence fragment with another fragment from the 3′ end of the same sequence in an inverted orientation. This involved two separate PCR reactions with a spacer or loop DNA in between. The transcripts produced from these constructs possess regions of self-complementarity, which can potentially form shRNA duplexes that generate siRNA within the cell, capable of degrading specific mRNA sequences. To amplify the sense sequence selected within the *GIP* gene’s ORF, specific primers were designed following the Tiscornia et al. protocol (Table 1) [18]. The forward primer included (1) an additional 4–5 nucleotides to facilitate digestion after PCR; (2) a recognition sequence for a 5′ restriction site to insert the PCR product into the pUC57 vector; and (3) approximately 20 nucleotides of the 5′ sense strand sequence. The reverse primer contained only 20 nucleotides that were complementary to the 3′ antisense strand sequence.

The PCR product was purified according to the procedure outlined in the commercial Wizard^®^ SV PCR Clean-Up System (Promega, Madison, WI, USA). This involved passing the sample through a chromatography column, where nucleic acids, along with a membrane binding solution, were retained on a silica membrane. Subsequently, the components of the PCR mix were removed using a washing solution. Finally, the DNA was eluted in nuclease-free water.

The ligation of the loop sequence (provided by Professor Sophien Kamoun at The Sainsbury Laboratory (TSL), Norwich, UK) was carried out using T4 DNA Ligase (Promega) following the manufacturer’s instructions.

For the amplification of the antisense sequence, a forward primer with approximately 20 nucleotides and a reverse primer were used, which included (1) an additional 4–5 nucleotides for digestion assistance; (2) a recognition sequence for a 3′ restriction site; (3) an AAAAA termination sequence in the complementary strand; and (4) 20 nucleotides that were complementary to the 3′ antisense strand sequence. The PCR product was purified as previously described and then ligated with T4 DNA Ligase (Promega) to the fragment containing the sense sequence and the loop.

The resulting construct, approximately 551 bp in length, was purified after ligation and subsequently digested with *Apa*I restriction enzyme in a final volume of 30 μL with a reaction buffer (1×) (Promega), 1.5 μL (7 U/μL) of ApaI enzyme, and 1 μg/μL DNA for 4 h at a temperature of 37 °C.

To estimate the concentration in comparison to the 500 bp band of the DNA ladder (Promega 100 bp ladder), the digested construct was visualized on a 1% agarose gel, excised with a sterile razor blade, and purified using the QIAquick^®^ Gel Extraction Kit following the manufacturer’s instructions (QIAGEN, Hilden, Germany). This purification was performed to enable direct ligation with the pUC57 vector, which had been previously digested by the ApaI restriction enzyme, using T4 DNA ligase (Promega).

### 3.4. Extraction of the Plasmid DNA of the Transformed E. coli

The bacterial colonies that had developed during the incubation phase and were anticipated to harbor recombinant plasmids were carefully selected and transferred into an LB liquid medium containing 100 μg/mL of ampicillin antibiotic. These cultures were then allowed to incubate overnight on a shaker set at 200 rpm, maintaining a temperature of 37 °C.

The plasmid miniprep was carried out employing the NZYMiniprep kit (NZYtech, Lisbon, Portugal) in adherence to the manufacturer’s guidelines. This method involved the alkaline lysis of bacterial cells, followed by the adsorption of DNA onto a silica surface. Subsequently, the recombinant plasmid was subjected to digestion using the ApaI restriction enzyme, after which it underwent agarose gel electrophoresis. The specific insert of interest was subsequently purified from the gel using the QIAquick^®^ Gel Extraction Kit, following the instructions provided by the manufacturer (QIAGEN, Hilden, Germany).

### 3.5. Cloning the Silencing Cassette into the pTH210 Vector

The pTH210 vector was obtained from *E. coli*, which had been previously transformed with it. The vector was then subjected to digestion using the ApaI enzyme, following the same procedure as described for the cassette digestion. Subsequently, a dephosphorylation step was conducted using Calf Intestinal Alkaline Phosphatase (CIAP). Dephosphorylation was essential to eliminate phosphate groups from the 5′ ends of the linearized plasmid, thus preventing recircularization. The linearized plasmid was treated with CIAP from Promega, USA, at a concentration of 0.01 UμL in the presence of 5 µL of the appropriate CIAP 10× buffer and ultra-filtered water to reach a final volume of 50 µL. This incubation occurred at 37 °C for 30 min.

Both the fragments of the silencing cassette and the pTH210 vector were excised from the gels using a sterile razor blade and subsequently purified by following the instructions provided by the QIAquick^®^ Gel Extraction Kit (QIAGEN, Hilden, Germany). Subsequently, the fragments were ligated together utilizing the T4 DNA Ligase enzyme.

To insert the cassette into the pTH210 vector, a ligation reaction was set up in a standard volume of 10 μL. This reaction contained DNA fragments with compatible ends, with 100 ng of plasmid used for a typical ligation reaction, along with 0.5 μL of T4 DNA ligase and 1 μL of ligation mix buffer (Promega). These reactions were left to incubate at 4 °C overnight. The resulting recombinant vector was employed for the transformation of *E. coli* NZY5 α competent cells. After plasmid extraction, further digestion with restriction enzymes was carried out to confirm the successful cloning of the cassette into the pTH210 vector.

### 3.6. Genomic Analysis of the Transformed Phytophthora cinnamomi (PCR and DNA Sequencing)

The mycelium of *P. cinnamomi*, which had been genetically transformed, was chosen as the source for extracting genomic DNA, followed by conducting a screening PCR to validate the integration of pTH210 into the genomic DNA of *P. cinnamomi*. The screening PCR was executed utilizing the primers listed in Table 1. Subsequently, the resulting amplification products were assessed through agarose gel electrophoresis. To confirm the integration, representative samples were forwarded to the Department of Microbiology and Genetics at the University of Salamanca. At this department, the samples underwent sequencing using the respective primers and an automated sequencer, namely the ABI PRISM 377W. This instrument was utilized to execute the electrophoretic separation and the detection of DNA fragments labeled with fluorescence markers.

### 3.7. Castanea sativa Infection with Phytophthora cinnamomi Strains

Infection of *C. sativa* roots was performed using mycelium from *P. cinnamomi* transformed and non-transformed to compare the effect of gene silencing on the plant phenotype. The roots were covered with fully colonized V8 medium, the plants were placed in sterile vermiculite, and they were incubated for 72 h at 25 °C.

### 3.8. Prediction of the Subcellular Localization of the GIP Protein

The software used to predict the protein localization was the following: SignalP 3.0 (accessible at http://www.cbs.dtu.dk/services/SignalP-3.0/), Cello (http://cello.life.nctu.edu.tw/), LOCtree (https://rostlab.org/services/loctree2/), EuK-mPLoc 2.0 (http://www.csbio.sjtu.edu.cn/bioinf/euk-multi-2/), and Esl pred (http://www.imtech.res.in/raghava/eslpred/index.html) (accessed on 14 September 2021).

## 4. Discussion

Our study aimed to investigate the effects of RNA interference using *P. cinnamomi* as a model organism due to its status as a serious pathogen that attacks various plants and causes significant economic losses. In this study, the RNA interference technique was applied using a shRNA-based vector to generate siRNA within the cell, which would degrade the mRNA sequence of the *GIP* gene. The potential effects on the plant phenotype were examined. The transformation with the *GIP*-silencing cassette had no observable effect on the phenotype and appearance of the transformed *P. cinnamomi* compared to their non-transformed counterparts. This is because the GIP protein is only produced during infection when it is secreted in the presence of the host [17].

The protein GIP is believed to undermine the effectiveness of the plant host’s surveillance system, leading to ink disease symptoms [16]. The selection of this gene was based on its critical role in countering plant defense mechanisms and inducing the development of disease symptoms [16]. Therefore, silencing this gene deprives the pathogen of an important weapon used to weaken the plant’s defense effectors, thereby facilitating the fight against the pathogen [1]. However, to date, no consideration has been given to potential interference between the *GIP*-silencing cassette and other similar genes, as a blast search revealed no similarities between *GIP* and other genes of *P. cinnamomi*.

The significance of RNA interference was recognized through research that produced a range of effective applications across various fields and purposes [19,20,21,22]. This technique has been explored in numerous agricultural studies as a promising tool for controlling pathogens and offering an environmentally friendly solution. While historically the transformation of *Phytophthora* protoplasts using polyethylene glycol and CaCl_2_ has been the method of choice, electroporation has been chosen in this study due to its rapid and efficient nature. Motile zoospores produced by *Phytophthora* species lack cell walls, which facilitate the transformation process [23].

In our study, the evaluation of plants’ phenotype after exposure to transformed *P. cinnamomi* carrying the silencing construct for three days revealed a lower percentage of disease development compared to plants infected with non-transformed *P. cinnamomi*, thus confirming the silencing of the *GIP* gene. These results serve as preliminary confirmation of the efficacy of RNA interference in silencing genes involved in the development of ink disease caused by *P. cinnamomi*.

Designing shRNAs that target specific genes to affect the proliferation of *P. cinnamomi* may be a viable method for limiting the spread of this pathogen and reducing its impact on plants, corroborating in this way with the study previously carried out by [1]. In general, achieving an effective knockout/knockdown method requires a careful selection of targets that result in a lethal phenotype.

## 5. Conclusions

In this study, we investigated the impact of pathogen gene silencing on the host phenotype using a shRNA-based vector capable of producing siRNA molecules that degrade the mRNA of the target gene. Our findings demonstrate that a silencing cassette for the *P. cinnamomi GIP* gene can be efficiently produced using a PCR-based cloning method, which offers a cost-effective alternative to other RNA interference mediators. Furthermore, the successful transformation of *P. cinnamomi* zoospores by electroporation was achieved, and the integration of the silencing construct into the genome was confirmed through genotypic tests, including PCR screening and sequencing.

To evaluate the effectiveness of the cassette, we infected C. sativa with transformed and non-transformed *P. cinnamomi* strains and observed the gene-silencing effect on the plant phenotype. Remarkably, chestnuts infected with transformed *P. cinnamomi* exhibited a lower percentage of wilting leaves and root necrosis compared to those infected with non-transformed *P. cinnamomi*. These results align with the known function of the *GIP* gene in triggering disease symptoms. Additionally, subcellular localization predictions revealed that the GIP protein is secreted through the classical ER-Golgi pathway. During plant infection, the pathogen secretes GIP proteins that bind to plant extracellular EGaseA, inhibiting its hydrolytic activity and thereby suppressing the induction of defense responses. The localization of the GIP protein at the plant extracellular EGaseA supports the prediction results.

This study highlights the potential of RNA interference as a promising approach to mitigate ink disease by targeting disease-related genes. Importantly, this method offers a non-controversial alternative to transgenic approaches for obtaining resistant crops.

As future objectives, we intend to characterize more genes involved in the infection processes of *Castanea sativa* crops and to produce more RNAi cassettes for silencing more genes. The use of RNAi cassettes to combat plant diseases can be a more ecological and sustainable alternative compared to the use of chemical pesticides. Reducing the susceptibility of plants to diseases can contribute to reducing the need to use chemical products to control pests. We still intend to analyze the effectiveness of the silencer cassette through RT-PCR and Next-Generation Sequencing (NGS). We believe this will allow for a more detailed functional analysis of the infection process and the elucidation of molecular communication mechanisms between the pathogen and host during infection. Additional research could be carried out, for example: (1) the transformation of *P. cinnamomi* with a vector housing various silencer cassette for different genes involved in the infection mechanism and the analysis of their effect on plant phenotype; (2) the analysis of how the silencing of certain genes impacts the plant proteome, examining the interaction between *P. cinnamomi* and *Castanea sativa* using the marking of proteins with different fluorescent markers.

## Figures and Tables

**Table 1:** List of primers used for *GIP*-silencing cassette construction.

Name	Sequence	Tm °C	GC%
FPCR1	5′GATAGGGCCCATGTGCACGACATGTTTACT3′	65.6	50
RPCR1	5′GCGACGGTGAAGACAACCGT3′	62.4	60
FPCR2	5′GCGACGGTGAAGACAACCGT3′	62.4	60
RPCR2	5′CTAGGGGCCCAAAAAAATGTGCACGACATGTTTACT3′	66.4	44.44

## Data Availability

All data generated or analyzed during this study are included in this published article (and its Supplementary Materials).

## Supplementary Materials

The following supporting information can be downloaded at https://www.mdpi.com/article/10.3390/plants12223821/s1, Figure S1. Alignment between the sequenced hygromycin PCR product and the *HPT* gene sequence. Figure S2. Alignment between the sequenced *GIP* silencing cassette and the *GIP* gene sequence.
