# Supplementary material for: Post-Transcriptional Gene Silencing of Glucanase Inhibitor Protein in Phytophthora cinnamomi
Source: Plants (Basel). 2023 Nov 10;12(22):3821. doi: 10.3390/plants12223821 (PMC10675509; doi:10.3390/plants12223821)
Supplement: Supplementary file 1 [file plants-12-03821-s001.zip › plants-2658983-supplementary.pdf]

# Supplementary material

**Post-Transcriptional Gene Silencing of Glucanase Inhibitor Protein in *Phytophthora cinnamomi***

## MATERIALS AND METHODS

### Sequencing of the transformed Phytophthora

To confirm the integration of the recombinant pTH210 vector in the genome of P. cinnamomi, the PCR products of the hygromycin fragment and the GIP silencing cassette were sequenced and then analyzed with the BioEdit program. After analysis with the BioEdit program, the nucleotide sequences were blasted using the NCBI database to investigate the ID of the sequenced genes. The alignment of similar genes was analysed and compared using the Muscle Tool Server (CLUSTAL multiple sequence alignment) from the EMBL-EBI database. The results of the alignment are represented in Figures S1 and S2.

```
CLUSTAL multiple sequence alignment by MUSCLE (3.8)


hpt_gene_sequence                     ATCGAAAAGTTCGACAGCGTCTCCGACCTGATGCAGCTCTCGGAGGGCGAAGAATCTCGT
hygromycin_fragment_clean_sequen      -----------------------------------GCTCTCGGAGGGCGAAGAATCTCGT
                                                                         *************************

hpt_gene_sequence                     GCTTTCAGCTTCGATGTAGGAGGGCGTGGATATGTCCTGCGGGTAAATAGCTGCGCCGAT
hygromycin_fragment_clean_sequen      GCTTTCAGCTTCGATGTAGGAGGGCGTGGATATGTCCTGCGGGTAAATAGCTGCGCCGAT
                                      ************************************************************

hpt_gene_sequence                     GGTTTCTACAAAGATCGTTATGTTTATCGGCACTTTGCATCGGCCGCGCTCCCGATTCCG
hygromycin_fragment_clean_sequen      GGTTTCTACAAAGATCGTTATGTTTATCGGCACTTTGCATCGGCCGCGCTCCCGATACCG
                                      ******************************************************** ***

hpt_gene_sequence                     GAAGTGCTTGACATTGGGGCATTCAGCGAGAGCCTGACCTATTGCATCTCCCGCCGTGCA
hygromycin_fragment_clean_sequen      GAAGTGCTTGACATTGGGGCATTCAGCGAGAGCCTTACCTATTGCATCTCCCGCCGTGCA
                                      *********************************** ************************

hpt_gene_sequence                     CAGGGTGTCACGTTGCAAGACCTGCCTGAAACCGAACTGCCCGCTGTTCTGCAGCCGGTC
hygromycin_fragment_clean_sequen      CAGGGTGTCACGTTGCAAGACCTGCCTGAAACCGAACTGCCCGCTGTTCTGCAGCCGGTC
                                      ************************************************************

hpt_gene_sequence                     GCGGAGGCCATGGATGCGATCGCTGCGGCCGATCTTAGCCAGACGAGCGGGTTCGGCCCA
hygromycin_fragment_clean_sequen      GCGGAGGCCATGGATGCGATCGCTGCGGCCGATCTTAGCCAGACGAGCGGGTTCGGCCCA
                                      ************************************************************

hpt_gene_sequence                     TTCGGACCGCAAGGAATCGGTCAATACACTACATGGCGTGATTTCATATGCGCGATTGCT
hygromycin_fragment_clean_sequen      TTCGGACCGCAAGGAATCGGTCAATACACTACATGGCGTGATTTCATATGCGCGATTGCT
                                      ************************************************************

hpt_gene_sequence                     GATCCCCATGTGTATCACTGGCAAACTGTGATGGACGACACCGTCAGTGCGTCCGTCGCG
hygromycin_fragment_clean_sequen      GATCCCCATGTGTATCACTGGCAAACTGTGATGGACGACACCGTCAGTGCGTCCGTCGCG
                                      ************************************************************

hpt_gene_sequence                     CAGGCTCTCGATGAGCTGATGCTTTGGGCCGAGGACTGCCCCGAAGTCCGGCACCTCGTG
hygromycin_fragment_clean_sequen      CAGGCTCTCGATGAGCTGATGCTTTGGGCCGAGGACTGCCCCGAAGTCCGGCACCTCTTG
                                      ********************************************************* **

hpt_gene_sequence                     CACGCGGATTTCGGCTCCAACAATGTCCTGACGGACAATGGCCGCATAACAGCGGTCATT
hygromycin_fragment_clean_sequen      CACGCGGATTTCGGCTCCAACAATGTCCTGACGGACAATGGCCGCATAACAGCGGTCATT
                                      ************************************************************

hpt_gene_sequence                     GACTGGAGCGAGGCGATGTTCGGGGATTCCCAATACGAGGTCGCCAACATCTTCTTCTGG
hygromycin_fragment_clean_sequen      GACTGGAGCGAGGCGATGTTCGGGGATTCCCAATACGAGGTCGCCAACATCTTCTTCTGG
                                      ************************************************************

hpt_gene_sequence                     AGGCCGTGGTTGGCTTGTATGGAGCAGCAGACGCGCTACTTCGAGCGGAGGCATCCGGAG
hygromycin_fragment_clean_sequen      AGGCCGTGGTTGGCTTGTATGGAGCAGCAGACGCGCTAGTTCGAGCGGAGGCATCCGGAG
                                      ************************************** *********************

hpt_gene_sequence                     CTTGCAGGATCGCCGCGGCTCCGGGCGTATATGCTCCGCATTGGTCTTGACCAACTCTAT
hygromycin_fragment_clean_sequen      CTTGCAGGATCGCCGCGGCTCCGGGCGTATATGCTCCGCATTGGTCTTGACCAACTCTAT
                                      ************************************************************

hpt_gene_sequence                     CAGAGCTTGGTTGACGGCAATTTCGATGATGCAGCTTGGGCGCAGGGTCGATGCGACGCA
hygromycin_fragment_clean_sequen      CAGAGCTTGGTTGACGGCAATTTCGATGATGCAGCTTGGGCGCAGGGTCGATACGACGCA
                                      **************************************************** *******

hpt_gene_sequence                     ATCGTCCGATCCGGAGCCGGGACTGTCGGGCGTACACAAATCGCCCGCAGAAGCGCGGCC
hygromycin_fragment_clean_sequen      ATCGTCCGATCCGGAGCCGGGACTGTCGGGCGTACACAAATCGCCCGCAGAAGCGCGGCC
                                      ************************************************************

hpt_gene_sequence                     GTCTGGACCGATGGCTGTGTAGAAGTACTCGCCGATAGTGGAAACCGACGCCCCAGCACT
hygromycin_fragment_clean_sequen      GTCTGGACCGATGGCTGTGTAGAAGTACTCGCCGATAGTGGAAACCGACGCCCCAGCACT
                                      ************************************************************

hpt_gene_sequence                     CGTCCGAGGGCAAAGGAATAGAGTAGATGCCGACCGAACAAGAGCTGATTTCGAGAACGC
hygromycin_fragment_clean_sequen      CGTCCGAGGGCAAAGGAATAGAGTAGATGCCGACCGAACAAGAGCTGATTTCGAGAACGC
                                      ************************************************************

hpt_gene_sequence                     CTCAGCCAGCAACTCGCGCGAGCCTATGAAGGCAAATGCGAGAGAACGGCCTTACGCTTG
hygromycin_fragment_clean_sequen      CTCAGCCAGCAACTCGCGCGAGCTTATGAAGGCAAATGCGAGAGAACGGCCTTAC-----
                                      *********************** *******************************

hpt_gene_sequence                     GTGGCACAGTTCTCGTCCACAGTTCGCTAAGCTCGCTCGGCTGGGTCGCGGGAGGCCGGT
hygromycin_fragment_clean_sequen      ------------------------------------------------------------
```

**Figure S1**. Alignment between the sequenced hygromycin PCR product and the *HPT* gene sequence

```
CLUSTAL multiple sequence alignment by MUSCLE (3.8)


gip_gene_sequence               TTTTTGAAATTCAGTAGCAAATATGTGCACGACATGTTTACTTCCGGAAGGGCTAAACGT
cassette_clean_sequence         ----------------------------------------CTTCCGGAAGGGCTAAACGT
                                                                        ********************

gip_gene_sequence               TCAATCCAATGGGCGTTTGTGAACCGCTCTTTACTCTATCTAACGTCCACCTCCTCAAGC
cassette_clean_sequence         TCAATCCAATGGTCGTTTGTGAACCGCTCTTTACTCTATCTAACGTCAACCTCCTCAAGC
                                ************ ********************************** ************

gip_gene_sequence               AATGGTTATCCACCATTGCGGAATTTACCGCGCACTGCTCACTCCATTTCGCCATCTTCC
cassette_clean_sequence         AATCGTTATCCACCATTGCGGAATTTACCGCGCACTGCTCACTCCATTTCGCCATCATCC
                                *** **************************************************** ***

gip_gene_sequence               GCTAACCATGACGGTTGTCTTCACCGTCGCCACTGCACTCATTGTGGTGGTGCTCTCATC
cassette_clean_sequence         GCTAACCATGACGGTTGTCTTCTCCGTCG-------------------------------
                                ********************** ******

gip_gene_sequence               AGTCCCACCCACGTGCTTACCACTGCGTCATGCACGGCATACGAGGAGGGCTCGTCCATC
cassette_clean_sequence         ----------------------------CGTGAAGGGTAAGCCAAGA-------------
                                                            * ** * ** *  * * **

gip_gene_sequence               CCCCACTGGGCTGCAGTGGGCACGCACTACATCAACGGCGCGAAAGATGGCGAGCGGATC
cassette_clean_sequence         ---CATTTGACTTC------------------------CACAAAAG--------------
                                   ** * * ** *                        * * ****

gip_gene_sequence               AAGATCGTGTCGACCAAGAACCACACACTATACAACTCGAGCAGTTTCTCGTACAATTTC
cassette_clean_sequence         --------------TAAGAGT-------------ACTCACGCA-----------------
                                               ****               ****  ***

gip_gene_sequence               GCTGTGTTGACACTTGAGAATCCAAGCAAGTTCGCCCCCGTCAAGCTCCCTAAGGCGGAT
cassette_clean_sequence         -CCACGCTGGCA-----------------------CCCAGTTGA----------------
                                 *   * ** **                       *** **  *

gip_gene_sequence               GGCTCGGACATTTTCCCGCGCGGTGGTCGAAGGTTATGTCGCTGGGGTGATACCAGCTAC
cassette_clean_sequence         --------------TCGGCGCG--------AGATTTAATCGCCG----------------
                                               * *****        ** **   **** *
```

**Figure S2**. Alignment between the sequenced *GIP* silencing cassette and the *GIP* gene sequence.

The alignment results of the hygromycin fragment sequence showed a high identity with the corresponding gene, while for the *GIP* cassette sequence, only the beginning of the sequence corresponding to the sense strand of the silencing construct showed a complete homology with the *P. cinnamomi GIP* gene. Because the construction of the cassette was based only on the sense sequence selected within the ORF of the *GIP* gene sequence, the antisense sequence is inverted compared with the gene sequence, and the loop does not have any homology with the *GIP* gene sequence. These results confirm that the silencing cassette has been integrated into the genome of *P. cinnamomi.*
